# Parents’ Diet Quality and Physical Activity Are Associated with Lifestyle in Spanish Children and Adolescents: The PASOS Study

**DOI:** 10.3390/nu15163617

**Published:** 2023-08-17

**Authors:** Margalida Monserrat-Mesquida, Marina Ródenas-Munar, Santiago F. Gómez, Julia Wärnberg, María Medrano, Marcela González-Gross, Narcís Gusi, Susana Aznar, Elena Marín-Cascales, Miguel A. González-Valeiro, Lluís Serra-Majem, Susana Pulgar, Marta Segu, Montse Fitó, Genís Según, Juan Carlos Benavente-Marín, Idoia Labayen, Augusto G. Zapico, Jesús Sánchez-Gómez, Fabio Jiménez-Zazo, Pedro E. Alcaraz, Marta Sevilla-Sánchez, Estefanía Herrera-Ramos, Helmut Schröder, Josep A. Tur, Cristina Bouzas

**Affiliations:** 1 Research Group on Community Nutrition and Oxidative Stress, University of Balearic Islands-IUNICS & IDISBA, 07122 Palma de Mallorca, Spain; 2 Centro de Investigación Biomédica en Red Fisiopatología de la Obesidad y la Nutrición (CIBEROBN), Institute of Health Carlos III, 28029 Madrid, Spain; 3 Health Research Institute of the Balearic Islands, 07120 Palma de Mallorca, Spain; 4 Gasol Foundation Europe, 08830 Sant Boi de Llobregat, Spain; 5 GREpS, Health Education Research Group, Nursing and Physiotherapy Department, University of Lleida, 25003 Lleida, Spain; 6 Epi-Phaan Research Group, Institute of Biomedical Research of Malaga (IBIMA), Universidad de Málaga, 29016 Málaga, Spain; 7 ELIKOS Group, Institute for Sustainability and Food Chain Innovation (IS-FOOD), Department of Health Sciences, Public University of Navarre, 31006 Pamplona, Spain; 8 ImFINE Research Group, Department of Health and Human Performance, Universidad Politécnica de Madrid, 28040 Madrid, Spain; 9 Physical Activity and Quality of Life Research Group (AFYCAV), Faculty of Sport Sciences, University of Extremadura, 06006 Cáceres, Spain; 10 PAFS Research Group, Faculty of Sports Sciences, University of Castilla-La Mancha-Toledo Campus, 45004 Toledo, Spainfabio.jimenez@uclm.es (F.J.-Z.); 11 UCAM Research Center for High Performance Sport, Universidad Católica de Murcia, 30107 Murcia, Spain; 12 Faculty of Sport Sciences, Universidad Católica de Murcia, 30107 Murcia, Spain; 13 Faculty of Sports Sciences and Physical Education, Universidade da Coruña, 15701 A Coruña, Spain; 14 Research Institute of Biomedical and Health Sciences (IUIBS), University of Las Palmas de Gran Canaria, 35001 Las Palmas, Spain; 15 Preventive Medicine Service, Centro Hospitalario Universitario Insular Materno Infantil (CHUIMI), Canarian Health Service, 35016 Las Palmas, Spain; 16 Regional Unit of Sports Medicine of Principado de Asturias, Municipal Sports Foundation of Avilés, 33402 Avilés, Spain; susana.pulgar@uneatlantico.es; 17 FC Barcelona Foundation, 08028 Barcelona, Spain; 18 Cardiovascular Risk and Nutrition Research Group (CARIN), Hospital del Mar Institute for Medical Research, 08003 Barcelona, Spain; 19 University of Lleida, 25003 Lleida, Spain; 20 Department of Didactics of Language, Arts and Physical Education, Universidad Complutense de Madrid, 28040 Madrid, Spain; 21 CIBER de Epidemiología y Salud Pública (CIBERESP), Instituto de Salud Carlos III, 28049 Madrid, Spain

**Keywords:** Mediterranean Diet, physical activity, exercise, lifestyle, kids, teenagers, PASOS

## Abstract

Background: Non-communicable chronic diseases are associated with a low-quality diet, low physical activity, and sedentary behavior. Objective: To assess how parents’ diet and physical activity habits were associated with their offsprings’ lifestyles. Study design: A cross-sectional analysis of 8–16-year-old children and adolescents (n = 2539; 51.9% girls) was carried out within the frame of the first edition of the Physical Activity, Sedentarism, Lifestyles, and Obesity in Spanish Youth study (PASOS-2019). Data on adherence to the Mediterranean Diet (MedDiet), daily moderate–vigorous physical activity (MVPA), and screen time per day (television, computer, video games, and mobile phone) were collected from children and adolescents, and data on parents’ diet quality and physical activity were compiled. Logistic regression models were used to assess the association between parents’ lifestyles and those of children and adolescents. Results: High diet quality of parents was associated with higher adherence to the MedDiet of children and adolescents, as well as high consumption of fruit, vegetables, fish, nuts, and legumes. The high physical activity level of parents was associated with the low consumption of fast foods, sweets, and candies in children and adolescents. Children with high levels of physical activity were those whose parents showed better diet quality and physical activity levels. Conclusions: Parents’ high diet quality and physical activity were associated with healthy lifestyles, higher adherence to the MedDiet, and physical activity of their offspring, mainly in adolescents.

## 1. Introduction

Non-communicable chronic diseases, such as obesity, type 2 diabetes, hypertension, dyslipidemia, insulin resistance, and psychological problems, were associated with low-quality diet, low physical activity, and sedentary behavior across the lifespan [1,2,3,4,5,6]. The Mediterranean Diet (MedDiet) was described as a dietary pattern useful to prevent non-communicable chronic diseases [7,8]. It was evidenced that the MedDiet has anti-inflammatory effects and high dietary antioxidant content, so adherence to the MedDiet is related to a better-quality diet [9,10]. It was demonstrated in 8–12-year-old children that a low level of physical activity was related to a poor-quality diet, especially low adherence to the MedDiet [11,12]. Children categorized as overweight and obese spent less time doing moderate-to-vigorous physical activity (MVPA) than their normal-weight peers [13]. Previous studies described that screen time activities, such as television, mobile telephones, computers, and video games, were associated with unhealthy lifestyle patterns [14,15] and insufficient sleep in children, promoting obesity in children and adolescents [16,17].

Parents could influence diet quality and behavioral habits in children [18,19]. It was pointed out that MVPA in children was related to parental support for children and parents’ sport activities [20]. A mother’s healthy lifestyle was associated with a decreased risk of obesity in children, showing the importance of multifactorial intervention in parents to decrease the risk of childhood obesity [21]. Lower parents’ educational levels were also related to unhealthy lifestyles and poor adherence to the MedDiet in Spanish children and adolescents [22]. Poor eating habits and high use of screen time in Spanish children were also associated with the lowest maternal educational level [23]. Eating patterns of children were associated with maternal eating and sedentary habits [24]. Relationships between parents’ diet and lifestyle and those of their children and adolescents were scarcely described in European countries, but not in Spain yet [25,26,27].

Therefore, the aim of the current study was to assess how parents’ diet and physical activity habits were associated with their offspring’s lifestyles.

## 2. Methods

### 2.1. Study Design

This study was a cross-sectional analysis within the frame of the first edition of the Physical Activity, Sedentarism, and Obesity in Spanish Youth (PASOS-2019) study, which was an observational, nationwide representative, and multicenter study. The design and methodology of the PASOS study were previously described [28].

### 2.2. Participants, Recruitment, and Ethics

Children and adolescents (8–16 years old; n = 3607) were recruited from March 2019 to February 2020 in 244 primary and secondary schools across the 17 Spanish autonomous communities. Each case was evaluated with teachers and parents or legal guardians, and individuals with an intellectual disability who could not respond to lifestyle questionnaires or had missing data were excluded (n = 1068). The final sample was 2539 children and adolescents.

The trial was registered in 2019 at the International Standard Randomized Controlled Trial registry (ISRCT; https://doi.org/10.1186/ISRCTN34251612 with the number 34251612). The PASOS study followed the Declaration of Helsinki ethical standards, and all procedures were approved according to the Ethics Committee of the Fundació Sant Joan de Déu, Barcelona, Spain (ref. PIC-179–18). All parents or legal guardians of each participant were informed of the purpose and the implications of the study, and all provided written informed consent, as well as all youth assent for participation.

### 2.3. Anthropometrics

Anthropometrics from the children/adolescents were measured by well-trained technicians according to the WHO standardized protocol [29]. Measurements of body weight and height were taken of children and adolescents in light clothing and without shoes, using an electronic SECA 899 scale (to the nearest 100 g) and a portable SECA 217 stadiometer (to the nearest 1 mm). Waist circumference measurements were taken, with the participants standing, by placing the tape just above the hipbones, putting the tape horizontal around the waist, not compressing the skin, and taking the measurement just after the participant breathed out, using a flexible non-stretch SECA 201 metric tape (to the nearest 1 mm). The BMI was calculated as body weight (kg)/height (m^2^). The BMI z-score was compared with the WHO 2007 growth standards for children and adolescents [30], according to the BMI z-score age and sex cut-offs: overweight and obese >1 standard deviation (SD); normal-weight (NW) ≥ −2 SD and ≤1 SD; and underweight < −2 SD.

### 2.4. Assessment of Mediterranean Diet Adherence and Diet Quality

A validated 16-item KIDMED questionnaire [31] was used to know the usual food consumption of the children and adolescents. The KIDMED index [32] was created to estimate adherence to the MedDiet in childhood, based on the principles that sustain MedDiet patterns, and designed with dichotomous response options (Yes/No). Items related to lower adherence were scored −1 (4 items) and items denoting higher adherence received +1 (12 items). This index was used to classify the subjects into three groups according to their adherence to the MedDiet: “low” = −4 to 3 points; “moderate” = 4 to 7 points; and “optimal” = 8 to 12 points.

Parents and legal guardians were asked to answer the Short Diet Quality Screener, which is a questionnaire on the frequency of consumption of 18 food groups [33,34]. Parents’ diet quality was classified into three categories which were created based on the tertile distribution of the resulting scores: “low diet quality”: <37.50; “medium diet quality”: 37.51–39.99; and “high diet quality”: >40.

### 2.5. Physical Activity and Sedentary Behavior

The physical activity of the children and adolescents was evaluated following the validated PAU-7S questionnaire [35] and categorized into two groups based on compliance with the MVPA daily recommendation (<60 min/day; ≥60 min/day) [36].

Parents’ physical activity was classified into three groups which were created based on the tertile distribution of the total Metabolic Equivalent of Task (MET) per day obtained in the sample of parents: “low physical activity”: <181.5 min/day; “medium physical activity”: 181.5–482.5 min/day; and “high physical activity”: >482.5 min/day. Children and adolescents were classified into three categories according to their parents’ diet quality and their parents’ physical activity.

Sedentary behavior in the youths was assessed by a validated screen-time sedentary behavior questionnaire [37] and categorized into two groups based on compliance with screen time recommendations from the American Academy of Pediatrics (<120 min/day; >120 min/day) [38].

### 2.6. Statistics

The Statistical Package for the Social Sciences version 27.0 (IBM SPSS Statistics for Windows, Chicago, IL, USA) was used to perform statistical analyses. Descriptive continuous variables, expressed as mean and standard deviation (SD), were analyzed by ANOVA. Descriptive categorical variables, expressed as sample size and percentage, were analyzed by χ^2^. The logistic regression analysis with the estimation of the corresponding odds ratio (OR) and the 95% confidence interval (CI) was calculated to assess the association between each child’s and adolescent’s affirmative answers in the KIDMED questionnaire (independent variables) with their parent’s diet quality and their parent’s physical activity (dependent variables), and to assess the association between each child’s and adolescent’s physical activity and screen time (independent variables) with their parent’s diet quality and their parent’s physical activity (dependent variables). In the parents’ diet quality classification, logistic regression analyses were adjusted for sex and body mass index (BMI) z-score because no differences between ages were found, whereas in the parents’ physical activity classification, logistic regression analyses were adjusted for age, sex, and body mass index (BMI) z-score. Results were considered significant when *p*-value < 0.05.

## 3. Results

For the first time, the results show how diet quality and physical activity of parents have an influence on children’s and adolescents’ lifestyles in Spain. Table 1 shows the characteristics of children according to their parents’ diet quality and physical activity. Youths presented more frequently with normal weight when their parents had a high diet quality and high physical activity.

Table 2 shows the association between significant children and adolescent KIDMED categories and parents’ diet quality. Parents’ diet quality was associated with the consumption of dairy products for breakfast and daily use of olive oil in children. The high diet quality of parents was positively associated with the consumption of two pieces of fruit, dairy, and raw or cooked vegetables per day; regular weekly fish consumption; and legumes consumption in adolescents. Parents’ high diet quality was significantly related to the optimal KIDMED index.

Table 3 shows the association between physical activity and screen time of offspring and the categories of their parents’ diet quality. High and medium diet quality of parents was significantly associated with meeting the daily recommended MVPA only in adolescents. A non-significant association was found regarding the achievement of the daily-use-of-screens recommendation.

Table 4 shows the association between the significant KIDMED categories of children and adolescents and their parents’ physical activity. Medium and high physical activity of parents was positively associated with the consumption of fruits and vegetables more than once a day, yogurts and/or 40 g cheese daily, and nuts regularly, as well as the use of olive oil at home in adolescents. Medium physical activity was positively associated with the consumption of raw or cooked vegetables daily also in adolescents. Moreover, medium and high physical activity of parents was related to a lower frequency of fast-food intake among adolescents, as well as lower sweets intake in children and lower consumption of rice or pasta than offspring of parents that are less active.

Table 5 shows the association between physical activity and screen time of offspring and the categories of their parents’ physical activity. High and medium physical activity of parents was associated with meeting the daily recommended MVPA and screen time on weekdays in offspring.

## 4. Discussion

The main findings of this study were that low parents’ diet quality and physical activity levels were related to low adherence to the MedDiet, and hence to a low diet quality, among Spanish youth, particularly in adolescents.

Previous studies pointed out that low adherence to the MedDiet in Spanish children and adolescents may be due to parents’ unhealthy lifestyles [39,40,41,42]. Regular consumption of dairy, nuts, and olive oil, as well as eating breakfast regularly, mainly with the parents, increases the quality of diet in adolescents [43]. The prevalence of nut consumption has become an ongoing health issue among adolescents [44], and moderate consumption of nuts during infancy could prevent an allergy to nuts [45]. It was also reported that the consumption of dairy products seems to be beneficial in muscle building, lowering blood pressure and low-density lipoprotein cholesterol, and also for preventing diabetes, tooth decay, obesity, and cancer [46]. The consumption of olive oil, especially extra-virgin olive oil, contributes to decreasing systolic blood pressure and improving endothelial function, cardiovascular disease, inflammation, and microbiota [47,48]. Current results are in accordance with these previous reports, since regular dairy, nut, and olive oil consumption, as well as eating breakfast regularly, occurred at a higher rate in the studied children and adolescents with parents with high diet quality. Therefore, it could be inferred that contemporary children with parents following a healthy diet show healthier habits and lifestyles.

When the relationship between the studied children/adolescents’ physical activity and their parents’ diet quality was assessed, it was found that adolescents whose parents followed a healthier diet were more likely to achieve the recommendation of a minimum of 60 min/day of MVPA, which is useful in preventing children from being categorized as overweight or obese [49]. Previous findings also demonstrated that intakes of fruits, vegetables, nuts, and olive oil in adolescents with parents with high physical activity were higher than those with parents with low physical activity [25,50]. A significant decrease in going to fast food restaurants was observed in adolescents whose parents performed high levels of physical activity, compared to those whose parents did only low physical activity [51]. Adolescents whose parents presented a high physical activity level showed a significant increase in physical activity practice and a decrease in screen time [52]. Current results are in accordance with these previous reports, confirming that parents play an important role in the early years of their children’s lifestyle, particularly in their physical activity and/or sedentary behavior, and that improving parents’ physical activity may also be a promising approach to implementing a healthy lifestyle in children and adolescents.

Positive associations between parents’ diet and parents’ physical activity with their children could be possible because there is evidence that parents influence their children’s behavior [25,50,51,52]. It has been shown that mothers with high education levels were associated with children’s healthy habits and low screen time [11,19]. The current results also showed that parental physical activity could have a positive impact on their children’s diet quality due to the fact children tend to have similar conduct to their parents, such as similar eating and exercise patterns [53]. Thus, it could be possible that these parents who did more physical activity also ate better and, accordingly, their children showed better diet quality.

The current study confirms the need to raise awareness among parents on the importance of following healthy lifestyles at home, aiming to improve their children’s or adolescents’ lifestyle and related health, as well as decreasing the risk of obesity and other non-communicable chronic diseases in children and adolescents.

## 5. Strengths and Limitations

This study has several strengths: Firstly, it followed a large representative sample of Spanish children and adolescents, including relevant factors associated with their parents’ histories, which were not frequently included in previous research. The current study also has several limitations. Firstly, causality for the significant associations cannot be established because the PASOS study was cross-sectional. Secondly, the food frequency questionnaire was not specific for children and adolescents; however, the KIDMED test is the most commonly used index of adherence to the MedDiet in the pediatric literature, allowing comparison among children and adolescents. Thirdly, a total of 1068 participants were dismissed because they were individuals with an intellectual disability who could not respond to lifestyle questionnaires or had missing data from the parents; however, since 2539 children and adolescents were recruited and followed, data are representative of Spanish children and adolescents.

## 6. Conclusions

A healthier lifestyle among parents is related to a healthy lifestyle, higher adherence to the MedDiet, and physical activity of their offspring, especially during adolescence. Adolescents are less prone to acquire detrimental social peers’ influences on their lifestyle if their parents follow a healthy lifestyle. Even though this was an observational study, it seems logical that healthy habits should be followed by parents to have an impact on their offspring’s lifestyle. Therefore, it is necessary to promote a healthy lifestyle among parents to improve healthy behaviors among children and adolescents.

## Figures and Tables

**Table 1:** Characteristics of children according to parents’ diet quality and physical activity.

	Parents’ Low Diet Quality (n = 880)	Parents’ Medium Diet Quality (n = 605)	Parents’ High Diet Quality (n = 1054)	*p*-Value
Age (mean, SD)	12.3 (2.4)	12.3 (2.3)	12.4 (2.3)	0.777
Boys (n; %)	447 (50.9)	279 (46.2)	538 (51.0)	0.387
Weight Status				
Underweight (n; %)	76 (9.1)	76 (12.6)	94 (8.9)	0.044
Normal weight (n; %)	499 (56.5)	319 (52.7)	651 (61.8)	
Overweight (n; %)	305 (34.4)	210 (34.7)	309 (29.3)	
Parents’ education level				
University degree (n; %)	293 (33.3)	210 (34.8)	421 (39.9)	0.019
General Certificate of Education (n; %)	164 (18.6)	116 (19.1)	190 (18.0)	
Vocational Education and Training (n; %)	201 (22.9)	148 (24.4)	201 (19.2)	
General Certificate of Secondary School (n; %)	120 (13.6)	43 (7.0)	114 (10.8)	
Primary Education (n; %)	97 (11.0)	83 (13.8)	114 (10.8)	
No education (n; %)	5 (0.6)	5 (0.9)	14 (1.3)	
	**Parents’** **Low Physical** **Activity** **(n = 840)**	**Parents’** **Medium Physical Activity** **(n = 848)**	**Parents’** **High Physical Activity** **(n = 851)**	***p*-Value**
Age (mean, SD)	12.4 (2.4)	12.6 (2.4)	12.7 (2.3) a	0.006
Boys (n; %)	402 (47.8)	406 (47.9)	412 (48.4)	0.965
Weight Status				
Underweight (n; %)	61 (7.3)	81 (9.6)	67 (7.9)	0.017
Normal weight (n; %)	442 (52.6)	485 (57.2)	493 (57.9)	
Overweight (n; %)	337 (40.1)	282 (33.2)	291 (34.2)	
Parents’ education level				
University degree (n; %)	222 (26.4)	308 (36.4)	227 (26.6)	<0.001
General Certificate of Education (n; %)	138 (16.5)	162 (19.1)	166 (19.6)	
Vocational Education and Training (n; %)	194 (22.9)	179 (21.0)	217 (25.5)	
General Certificate of Secondary School (n; %)	125 (14.8)	79 (9.3)	115 (13.5)	
Primary Education (n; %)	144 (17.3)	108 (12.8)	114 (13.4)	
No education (n; %)	17 (2.1)	12 (1.4)	12 (1.4)	

Weight status was categorized by body mass index (BMI) z-score. Cut-off values for weight status were ±1 SD. *p*-value for the chi-square or ANOVA test among parents’ physical activity and the variables studied. Different letters in rows show statistically significant differences between categories of physical activity by the Bonferroni post hoc test: ^a^ low physical activity vs. high physical activity.

**Table 2:** Association of significant children and adolescent KIDMED categories and parents’ diet quality.

	Parents’ Low Diet Quality (n = 880)	Parents’ Medium Diet Quality (n = 605)	Parents’ High Diet Quality (n = 1054)
Q2. Has dairy product for breakfast (+)	1.00 (ref.)	1.84 (1.16–2.93) *	1.57 (1.08–2.28) *
Children	1.00 (ref.)	2.74 (1.30–5.77) **	1.63 (0.84–2.82)
Adolescents	1.00 (ref.)	1.32 (0.72–2.42)	1.48 (0.88–2.78)
Q6. Takes a second serving of fruit daily (+)	1.00 (ref.)	1.66 (1.21–2.29) **	1.80 (1.37–2.37) ***
Children	1.00 (ref.)	1.46 (0.93–2.29)	1.50 (1.01–2.22) *
Adolescents	1.00 (ref.)	1.89 (1.19–3.02) **	2.22 (1.49–3.31) ***
Q7. Consumes yogurts and/or 40 g cheese daily (+)	1.00 (ref.)	1.37 (0.92–2.05)	1.40 (1.00–1.96)
Children	1.00 (ref.)	1.12 (0.66–1.88)	1.13 (0.72–1.78)
Adolescents	1.00 (ref.)	1.91 (1.00–3.65) *	1.83 (1.09–3.09) *
Q8. Consumes raw or cooked vegetables daily (+)	1.00 (ref.)	1.16 (0.83–1.61)	1.40 (1.05–1.86) *
Children	1.00 (ref.)	0.89 (0.56–1.42)	1.18 (0.78–1.79)
Adolescents	1.00 (ref.)	1.49 (0.93–2.40)	1.64 (1.10–2.44) *
Q9. Consumes raw or cooked vegetables more than 1/day (+)	1.00 (ref.)	1.13 (0.80–1.61)	1.68 (1.25–2.26) ***
Children	1.00 (ref.)	0.96 (0.59–1.56)	1.85 (1.23–2.78) **
Adolescents	1.00 (ref.)	1.36 (0.81–2.29)	1.55 (1.00–2.41) *
Q10. Regular fish consumption (at least 2–3 week) (+)	1.00 (ref.)	1.34 (0.96–1.87)	1.66 (1.24–2.21) ***
Children	1.00 (ref.)	1.35 (0.84–2.17)	1.43 (0.95–2.17)
Adolescents	1.00 (ref.)	1.30 (0.81–2.06)	1.88 (1.26–2.81) **
Q12. Regular nut consumption (≥2–3/week) (+)	1.00 (ref.)	1.03 (0.75–1.42)	1.43 (1.09–1.88) **
Children	1.00 (ref.)	0.99 (0.64–1.57)	1.39 (0.93–2.07)
Adolescents	1.00 (ref.)	0.97 (0.62–1.54)	1.41 (0.96–2.07)
Q13. Legumes more than 1/week (+)	1.00 (ref.)	1.18 (0.84–1.67)	1.57 (1.16–2.13) **
Children	1.00 (ref.)	1.04 (0.64–1.67)	1.51 (0.98–2.33)
Adolescents	1.00 (ref.)	1.31 (0.79–2.16)	1.58 (1.03–2.42) *
Q16. Uses olive oil at home (+)	1.00 (ref.)	1.76 (0.96–3.21)	1.48 (0.91–2.39)
Children	1.00 (ref.)	1.33 (1.06–5.13) *	1.35 (0.75–2.41)
Adolescents	1.00 (ref.)	1.17 (0.44–3.08)	1.84 (0.75–4.49)
KIDMED Index			
Low	1.00 (ref.)	0.50 (0.28–0.91) *	0.53 (0.33–0.86) **
Moderate	1.00 (ref.)	0.80 (0.58–1.10)	0.62 (0.47–0.82) **
Optimal	1.00 (ref.)	1.59 (1.15–2.20) **	2.01 (1.52–2.66) ***

Abbreviations: Q, question. Odds ratio (OR) with 95% confidence intervals (CI) was calculated by binary logistic regression analysis. The analysis was adjusted by sex and body mass index (BMI) z-score. *p*-value: * < 0.05; ** < 0.01; *** < 0.001.

**Table 3:** Association between offspring physical activity and screen time and categories of parents’ diet quality.

	Parents’ Low Diet Quality (n = 880)	Parents’ Medium Diet Quality (n = 605)	Parents’ High Diet Quality (n = 1054)
MVPA ≥ 60 min/day	1.00 (ref.)	1.23 (0.88–1.73)	1.45 (1.08–1.93) *
Children	1.00 (ref.)	0.86 (0.54–1.37)	1.17 (0.78–1.75)
Adolescents	1.00 (ref.)	1.80 (1.08–2.98) *	1.85 (1.20–2.84) **
Total screen time on weekdays < 120 min/day	1.00 (ref.)	1.19 (0.87–1.64)	1.19 (0.91–1.57)
Children	1.00 (ref.)	1.10 (0.68–1.78)	1.33 (0.87–2.03)
Adolescents	1.00 (ref.)	1.23 (0.75–2.01)	1.15 (0.76–1.75)

Abbreviations: MVPA: moderate and vigorous physical activity; min: minute. Odds ratio (OR) with 95% confidence intervals (CI) was calculated by binary logistic regression analysis. The analysis was adjusted by sex and body mass index (BMI) z-score. *p*-value: * < 0.05; ** < 0.01.

**Table 4:** Association of significant children and adolescent KIDMED categories and parents’ physical activity.

	Parents’ Low Physical Activity (n = 840)	Parents’ Medium Physical Activity(n = 848)	Parents’ High Physical Activity (n = 851)
Q6. Takes a second serving of fruit daily (+)	1.00 (ref.)	1.08 (0.89–1.32)	1.28 (1–05−1.56) *
Children	1.00 (ref.)	1.13 (0.84–1.51)	1.23 (0.91–1.66)
Adolescents	1.00 (ref.)	1.09 (0.83–1.43)	1.43 (1.10–1.87) **
Q7. Consumes yogurts and/or 40 g cheese daily (+)	1.00 (ref.)	1.26 (1.00–1.60)	1.27 (1.01–1.61) *
Children	1.00 (ref.)	1.21 (0.87–1.69)	1.08 (0.77–1.52)
Adolescents	1.00 (ref.)	1.31 (0.94–1.81)	1.44 (1.04–2.0) *
Q8. Consumes raw or cooked vegetables daily (+)	1.00 (ref.)	1.08 (0.88–1.32) *	1.08 (0.88–1.32)
Children	1.00 (ref.)	1.19 (0.88–1.62)	1.05 (0.77–1.43)
Adolescents	1.00 (ref.)	1.39 (1.05–1.84) *	1.10 (0.84–1.45)
Q11. Goes >1/week fast food restaurant (−)	1.00 (ref.)	0.78 (0.62–0.98) *	0.70 (0.56–0.89) **
Children	1.00 (ref.)	0.81 (0.58–1.15)	0.72 (0.50–1.03)
Adolescents	1.00 (ref.)	0.75 (0.55–1.03)	0.69 (0.50–0.95) *
Q12. Regular nut consumption (≥2–3/week) (+)	1.00 (ref.)	1.20 (0.98–1.46)	1.27 (1.04–1.54) *
Children	1.00 (ref.)	1.16 (0.87–1.55)	1.26 (0.93–1.70)
Adolescents	1.00 (ref.)	1.25 (0.96–1.63)	1.32 (1.01–1.72) *
Q14. Takes sweets and candies several times every day (−)	1.00 (ref.)	0.83 (0.65–1.06)	0.75 (0.59–0.96)
Children	1.00 (ref.)	0.63 (0.43–0.91) *	0.74 (0.51–1.07)
Adolescents	1.00 (ref.)	1.00 (0.73–1.38)	0.76 (0.55–1.05)
Q15. Consumes rice or pasta almost daily (≥5/week) (+)	1.00 (ref.)	0.78 (0.64–0.95) *	0.99 (0.82–1.21)
Children	1.00 (ref.)	0.69 (0.52–0.92) *	0.89 (0.66–1.20)
Adolescents	1.00 (ref.)	0.87 (0.66–1.14)	1.08 (0.83–1.41)
Q16. Uses olive oil at home (+)	1.00 (ref.)	1.04 (0.89–1.83)	1.04 (0.74–1.48)
Children	1.00 (ref.)	0.96 (0.62–1.47)	0.86 (0.56–1.33)
Adolescents	1.00 (ref.)	2.42 (1.17–5.00) *	1.24 (0.69–2.24)

Abbreviations: Q, question. (+): positive value on KIDMED score; (−): negative value on KIDMED score. Odds ratio (OR) with 95% confidence intervals (CI) was calculated by binary logistic regression analysis. The analysis was adjusted by age, sex, and body mass index (BMI) z-score. *p*-value: * < 0.05; ** < 0.01.

**Table 5:** Association between offspring physical activity and screen time and categories of parents’ physical activity.

	Parents’ Low Physical Activity (n = 840)	Parents’ Medium Physical Activity (n = 848)	Parents’ High Physical Activity (n = 851)
MVPA ≥ 60 min/day	1.00 (ref.)	0.92 (0.75–1.14)	1.25 (1.02–1.54) *
Children	1.00 (ref.)	0.86 (0.54–1.37)	1.17 (0.78–1.75)
Adolescents	1.00 (ref.)	1.09 (0.80–1.49)	1.65 (1.66–2.24) **
Total screen time on weekdays < 120 min/day	1.00 (ref.)	1.22 (1.00–1.48)	1.13 (0.92–1.37)
Children	1.00 (ref.)	1.39 (1.02–1.90) *	1.35 (0.98–1.85)
Adolescents	1.00 (ref.)	1.24 (0.92–1.66)	1.21 (0.91–1.62)

Abbreviations: MVPA: moderate and vigorous physical activity; min: minute. Odds ratio (OR) with 95% confidence intervals (CI) was calculated by binary logistic regression analysis. The analysis was adjusted by age, sex, and body mass index (BMI) z-score. *p*-value: * < 0.05; ** < 0.01.

## Data Availability

There are restrictions on the availability of data for this trial, due to the signed consent agreements around data sharing, which only allow access to external researchers for studies following the project purposes. Requestors wishing to access the trial data used in this study can make a request to pep.tur@uib.es.
